# Cytokine responses of human lung cells (BEAS-2B) treated with micron-sized and nanoparticles of metal oxides compared to soil dusts

**DOI:** 10.1186/1743-8977-4-2

**Published:** 2007-02-27

**Authors:** John M Veranth, Erin G Kaser, Martha M Veranth, Michael Koch, Garold S Yost

**Affiliations:** 1 Department of Pharmacology and Toxicology, University of Utah, Salt Lake City, Utah, USA

## Abstract

**Background:**

The induction of cytokines by airway cells *in vitro *has been widely used to assess the effects of ambient and occupational particles. This study measured cytotoxicity and the release of the proinflammatory cytokines IL-6 and IL-8 by human bronchial epithelial cells treated with manufactured nano- and micron-sized particles of Al_2_O_3_, CeO_2_, Fe_2_O_3_, NiO, SiO_2_, and TiO_2_, with soil-derived particles from fugitive dust sources, and with the positive controls LPS, TNF-α, and VOSO_4_.

**Results:**

The nano-sized particles were not consistently more potent than an equal mass of micron-sized particles of the same nominal composition for the induction of IL-6 and IL-8 secretion in the *in vitro *models used in this study. The manufactured pure oxides were much less potent than natural PM_2.5 _particles derived from soil dust, and the cells were highly responsive to the positive controls. The nano-sized particles in the media caused artifacts in the measurement of IL-6 by ELISA due to adsorption of the cytokine on the high-surface-area particles. The potency for inducing IL-6 secretion by BEAS-2B cells did not correlate with the generation of reactive oxygen species in cell-free media.

**Conclusion:**

Direct comparisons of manufactured metal oxide nanoparticles and previously studied types of particles and surrogate proinflammatory agonists showed that the metal oxide particles have low potency to induce IL-6 secretion in BEAS-2B cells. Particle artifacts from non-biological effects need to be considered in experiments of this type, and the limitations inherent in cell culture studies must be considered when interpreting *in vitro *results. This study suggests that manufactured metal oxide nanoparticles are not highly toxic to lung cells compared to environmental particles.

## Background

Nanosized particles of many different metal and ceramic oxides are currently being commercially manufactured for applications that include high-performance composite materials, abrasives in semiconductor manufacturing, photochemically active or wavelength selective surface coatings, process catalysts, electronic components, and cosmetics. Particles smaller than 100 nm of carbon, silica and titanium dioxide are currently sold as bulk chemicals, and many more nanoparticle types will be produced in tonnage quantities as manufacturing technology improves and prices are reduced. Increased production volume on nanomaterials will lead to increased human and environmental exposure from normal use, fugitive emissions, accidental spills, and disposal of materials after use. Current environmental laws and occupational health guidelines are based on the nominal chemical composition and seldom specify special standards for ultrafine or nano-sized particles. The potential occupational health and environmental effects of these nano-sized powders are a public policy concern, and this research was funded by a program addressing toxicology of manufactured nanomaterials.

Particle-induced tissue inflammation has been proposed as a central process connecting inhaled particles with adverse health effects [1]. The onset and resolution of inflammation is regulated by cytokines, a class of signaling molecules associated with many processes including cell growth and differentiation, physiological responses of tissues, and recruitment of macrophages and other mobile cells to specific sites [2-4]. Cytokine assays are widely used in studies of lung cell responses to particles and other pollutants [5]. Much of the recent *in vitro *particle toxicology research has used ELISA assays to measure the cytokines IL-6 [6-8], IL-8 [9-11], and TNF-α [12-14] in cell cultures exposed to different types of solid particles.

There is increasing evidence suggesting that redox-active transition metals associated with particles can induce pro-inflammatory responses in lung cells [9,15-18]. Reactive oxygen species (ROS) is a non-specific term that includes both radicals (OH•, O_2 _^-^•) and non-radicals (H_2_O_2_). There is evidence for both extracellular and intracellular reactions leading to ROS formation in particle-treated *in vitro *systems [14,19,20]. Extracellular generation of ROS has been proposed as a mechanism by which particles may stimulate cell-surface receptors. The oxidation of the probe dichlorodihydrofluorescein (DCFH) to the fluorescent compound 2' – 7' dichlorofluorescein (DCF) is a common way of measuring ROS in cell cultures [21-23]. The DCFH assay measures multiple species including RO_2_•, RO•, OH•, HOCl, ONOO- [24] and the products of metal-catalyzed reactions involving peroxide.

Animal studies with carbon black and titanium dioxide particles reported that particles smaller than 30 nm have a greater ability to induce lung inflammation than larger particles with the same nominal composition [25,26]. Both Donaldson [27] and Oberdörster [28] concluded in reviews that ultrafine particles of low-solubility, low-toxicity materials are more inflammogenic in the rat lung than larger particles from the same material, and hypothesized that the effects are related to surface area and involve oxidative stress.

Although many studies have investigated particle-induced cytokine responses *in vitro*, it is difficult to make quantitative comparisons of various particle types from the literature. Comparisons are confounded by differences between studies in the biological model tested (rodent or human macrophages or epithelial cells, immortalized or normal cells), the experimental protocol (concentration, duration), and endpoints reported (cell death, cytokine secretion, changes in mRNA). This study used a consistent set of *in vitro *experimental protocols to study six different compositions of manufactured particles that are commercially available as both nano- and micron sized powders. We used a nominal diameter of 30 nm as the definition of nanosized particles and compared these to particles larger than 1 μm diameter of the same nominal chemical composition. We also compared the manufactured metal and ceramic oxide particles to soil-derived dusts [29,30] and to the positive controls lipopolysaccharide (LPS), tumor necrosis factor-alpha (TNF-α) and soluble vanadium (VOSO_4_). The objectives were to: 1) characterize the proinflammatory cytokine response of human lung epithelial cells treated *in vitro *with manufactured metal and ceramic oxide particles, 2) compare nano-sized and micron-sized particle pairs for several different oxides, and 3) compare the cytokine responses induced by manufactured oxide particles to the responses to natural minerals and surface-derived fugitive dusts.

## Results

### Particle characterization

The particles used in this study are described in Table 1. The surface-mean particle size calculated from nitrogen adsorption area measurement was close to the nominal particle size furnished by the vendor except for the supermicron particles of NiO. The adsorbtion surface area was much greater than the geometrical surface for the nominal diameter indicating that the NiO particles either are aggregates or have internal porosity. The natural dusts, prepared by resuspension and aerodynamic separation with an upper size cutpoint of <3 μm, also have high surface area indicating that the nominal PM_2.5 _dust had a broad size distribution and contained significant amounts of smaller particles.

**Table 1:** Particle characteristics.

Code	Name	Nominal Size μm	Vendor	Surface m^2^/g	Surface mean diameter μm	Comment
Al-N	nano Al_2_O_3_	0.008 – 0.014	3	261	0.006	alpha alumina
Al-M	micron Al_2_O_3_	1	1	7.7	0.21	gamma alumina
Ce-N	nano CeO_2_	0.009 – 0.015	1	71.3	0.014	
Ce-M	micron CeO_2_	5	1	0.6	1.5	
Fe-N	nano Fe_2_O_3_	0.003	1	221	0.005	
Fe-M	micron Fe_2_O_3_	5	1	11.6	0.10	
Ni-N	nano NiO	0.010 – 0.020	5	145	0.006	
Ni-M	micron NiO	< 10	5	56.9	0.016	
Si-N	nano SiO_2_	0.010	3	127	0.019	amorphous silica
Si-M	micron SiO_2_	0.5 – 10	1	5.4	0.44	amorphous silica
Ti-N	nano TiO_2_	0.005	3	242	0.006	anatase
Ti-M	micron TiO_2_	1 – 2	1	3.5	0.41	rutile
KLN	kaolin		2	24.3	0.1	commercial clay for ceramics
MUS	Min-U-Sil	< 5	6			ground crystalline silica
DD	rural soil, Desert	< 3	4	6.2	0.4	PM_2.5_-enriched natural dust
JE	urban street, Juarez	< 3	4			PM_2.5_-enriched natural dust
MNC	rural soil, Mancos	< 3	4	13.0	0.2	PM_2.5_-enriched natural dust
LPS	lipopoly-saccharide	soluble	5			positive control P. aeruginosa 1000 EU/mL
V	VOSO_4_	soluble	1			positive control 19 μg/mL
TNF-α	tumor necrosis factor alpha	soluble	5			positive control 0.01 μg/mL

Supplier codes: 1) Alfa Aesar; 2) Capitol Ceramics, Salt Lake City, UT; 3) Nanostructured and Amorphous Materials, Los Alamos NM; 4) Natural mixture, field sampling by authors; 5) Sigma Aldrich; 6) US Silica.

The elemental compositions of the natural soil-derived dusts as measured by x-ray fluorescence are dominated by the crustal elements, Al, Si, Ca, Mg, and Ti. The endotoxin content, organic carbon content (functionally defined as the carbon that is volatile in He atmosphere at temperature steps < 550°C) and the elemental carbon content (carbon that is removed in 2% O_2_/98% He at temperatures from 550 to 800°C) are listed in Table 2.

**Table 2:** Soil-derived particle composition.

Identification	Organic Carbon, %	Elemental Carbon, %	Endotoxin EU/mg
DD	8.1	0.26	27
JE	21.1	1.6	274
MNC	3.8	0.48	5.3

### Cell responses

**Figure 1:**
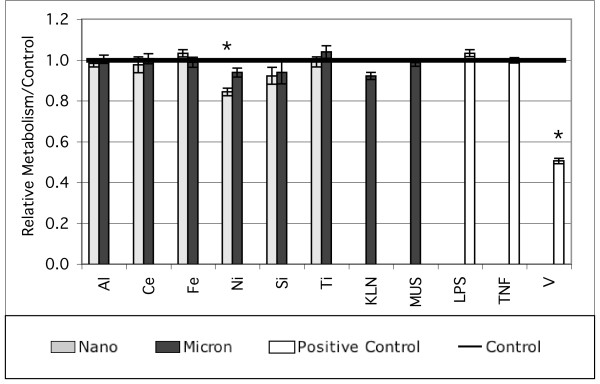
Treatments at the maximum particle concentrations used in this study were not highly toxic to the cells as indicated by a mitochondrial reductase assay. Data are mean ± s.d., normalized by the control, N = 9, and are typical of multiple cell passages. * designates statistically different than control. Sample ID codes are in Table 1.

Cell viability was evaluated to insure that low levels of cytokine secretion were not caused by cell death during the treatment period. Figure 1 shows the assay results for the highest treatment concentration (53 μg/cm^2^) of particles and for the concentrations of positive controls listed in Table 1. Only the VOSO_4 _positive control caused more than 20% loss of cell viability at the 24-h time point, as measured by the mitochondrial reduction of WST-8.

**Figure 2:**
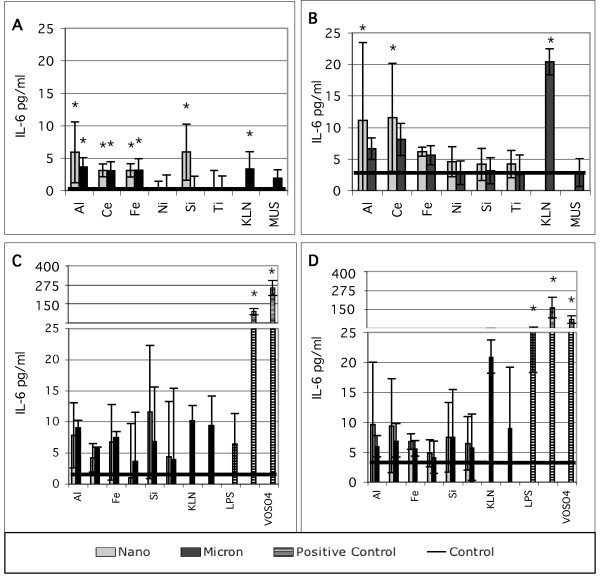
IL-6 response of *in vitro *cell cultures treated with equal mass concentrations (53 μg/cm^2^) of nano- and micron-sized particles of the same nominal substance. A and B represent single experiments, N = 9. C and D are merged results from two independent experiments, N = 9 and N = 4 and have a broken scale to show the positive controls. A, C, : BEAS-2B cells in KGM media with 0.1% BSA; B, D. BEAS-2B cells in LHC-9 media with 0.1% BSA, IL-6 concentrations in pg/mL, mean ± s.d. *denotes statistically greater than control. Treatment codes are in Table 1.

The release of IL-6 by the immortalized cell line BEAS-2B in response to treatment with pairs of nano- and micron-sized particles is shown in Figure 2. Experiments were conducted in both KGM (2a and 2c) and LHC-9 media (2b and 2d). Graphs in 2a and 2b show typical results obtained from single experiments. Presented in this way the data suggest that the metal oxide particles induce small increases in IL-6 secretion, but that the nano-sized particles are not consistently more potent than the larger particles in each pair. Graphs 2c and 2d present the data in 2a and 2b merged with a followup experiment and also present the results from positive controls run in both experiments. The agreement between the two experiments is good, but the larger data set suggests that the IL-6 response to all the pure oxide manufactured particles is small compared to the positive controls. The pure oxides were also compared to kaolin (pottery clay), and Min-U-Sil (commercial mechanically ground crystalline silica), and neither of these particle was highly potent for inducing IL-6 secretion. The experiments in Figure 2 were conducted 5.3 and 53 μg/cm^2 ^particle concentration in media with 0.1% BSA added. Data shown are for the high particle treatment concentration and for the positive control concentrations in Table 1. Similar small responses to pure oxide particles compared to positive controls were seen at the low dose and in experiments with as-formulated media.

**Figure 3:**
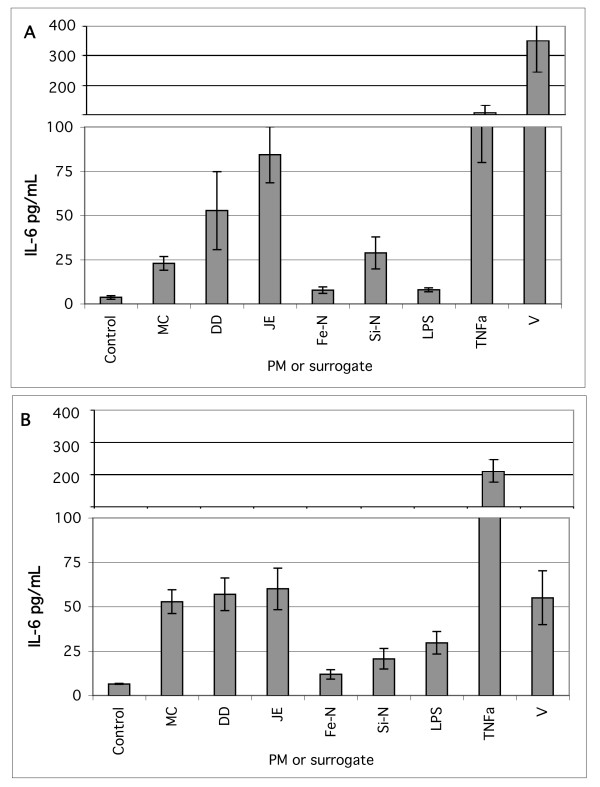
Comparison of manufactured Fe_2_O_3 _and SiO_2 _nanoparticles to soil-derived dusts (DD, JE, MC) at 53 μg/cm^2 ^and to positive controls at concentrations listed in Table 1. IL-6 concentration in pg/mL, mean ± standard error of the mean (95% confidence interval) based on 5 independent experiments. Note the discontinuity in the Y-axis scale. A. KGM media with BSA 0.1% BSA. B. LHC-9 with 0.1% BSA.

To further characterize the response of BEAS-2B cells to the manufactured oxide particles we conducted a separate set of experiments where each cell culture plate contained two of the nano-sized particle types, soil-derived particles from three different sites, and positive controls. Figure 3 shows the merged results of five independent experiments conducted in KGM and LHC-9 media with particles at 53 μg/cm2 and with the positive controls at concentrations listed in Table 1. The manufactured metal and ceramic oxide particles were less potent for the induction of IL-6 release than an equal mass concentration of three different soil-derived dusts. The LPS, TNF-a, and soluble vanadium are positive controls that show that the BEAS-2B cells are capable of producing IL-6 under the experimental conditions. The DD, JE, and MC samples are PM_2.5_-enriched dusts prepared from surface soil samples, and are the same materials used in our previous studies of cytokine response in epithelial cells exposed to atmospheric fugitive dust particles [29,30]. These data strengthen the conclusion that the IL-6 secretion response of BEAS-2B cells to the pure oxides is small compared to other previously studied and environmentally relevant particle types.

Nano-sized SiO_2 _particles caused a statistically significant increase in IL-6 compared to both the untreated control and the cells treated with micron-sized SiO_2 _particles in six consecutive experiments with BEAS-2B cells indicating a reproducible pattern. For the other nano- and micron-sized particle pairs there was no conclusive evidence for the nanoparticles being consistently more potent than an equal mass concentration of the paired micron-sized particles.

The relative responses of BEAS-2B cells in the two types of cell culture media are characteristic of what we observed in a separate study comparing different *in vitro *lung cell models for particle toxicology [manuscript in preparation]. BEAS-2B cells in KGM media are highly responsive to vanadium and other soluble metals, as has been reported by others [31-34], but show relatively low response to LPS. In LHC-9 media the relative response to LPS and soluble metals is reversed.

**Figure 4:**
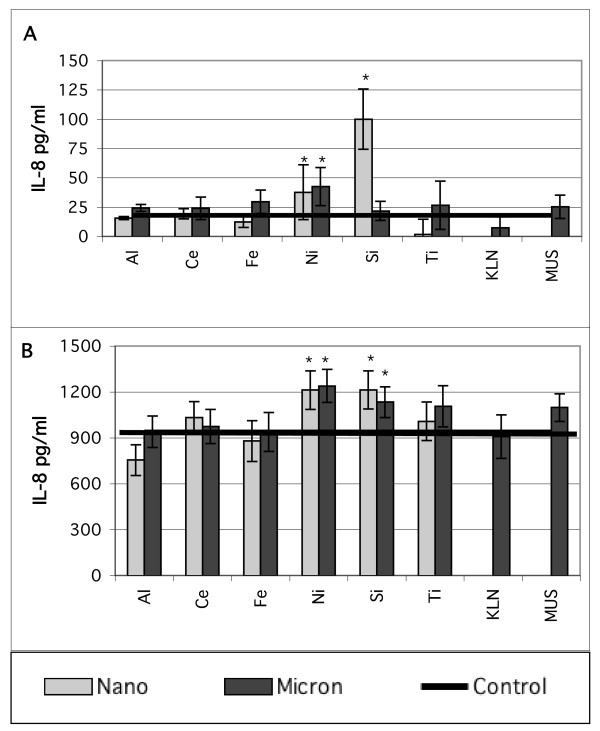
IL-8 response of *in vitro *cell cultures treated with equal mass concentrations (53 μg/cm^2^) of nano- and micron-sized particles of the same nominal substance. A. BEAS-2B cells in LHC-9 media with 0.1% BSA, B. NHBE cells in BEGM media with 0.1% BSA. IL-8 in pg/mL, mean ± s.d., N = 9, * denotes statistically greater than control. Treatment codes are in Table 1.

**Figure 5:**
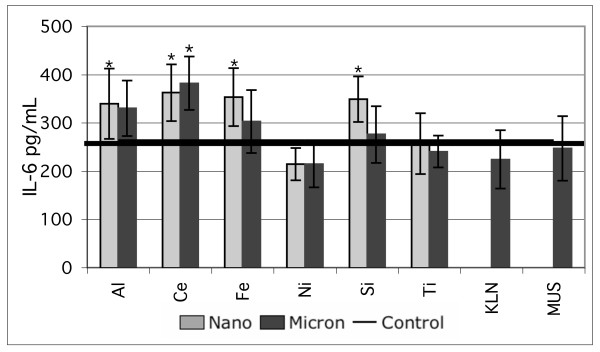
IL-6 response for a typical experiment with NHBE cells in BEGM media with 0.1% BSA treated at the same particle concentration as previous figures. IL-6 concentrations in pg/mL, mean ± s.d., N = 9, *denotes statistically greater than control. Treatment codes are in Table 1.

The focus of this study was on IL-6 secretion by BEAS-2B cells, but we also measured IL-8 secretion and tested other cell types. Figure 4 shows typical IL-8 secretion data from a typical experiment with treatment at the high particle concentation. Again, any responses to the pure metal oxides are small and near control levels, and similar small responses were also seen at the low treatment concentration. Figure 5 shows typical IL-6 secretion in normal human bronchial epithelial cells (NHBE, Clonetics). The NHBE cells produce much higher control levels of IL-6 than the BEAS-2B cells, but the increased secretion in response to the pure oxide particles is small for both the 53 and 5.3 μg/cm^2 ^particle treatmetn concentrations.

### Particle artifacts

**Figure 6:**
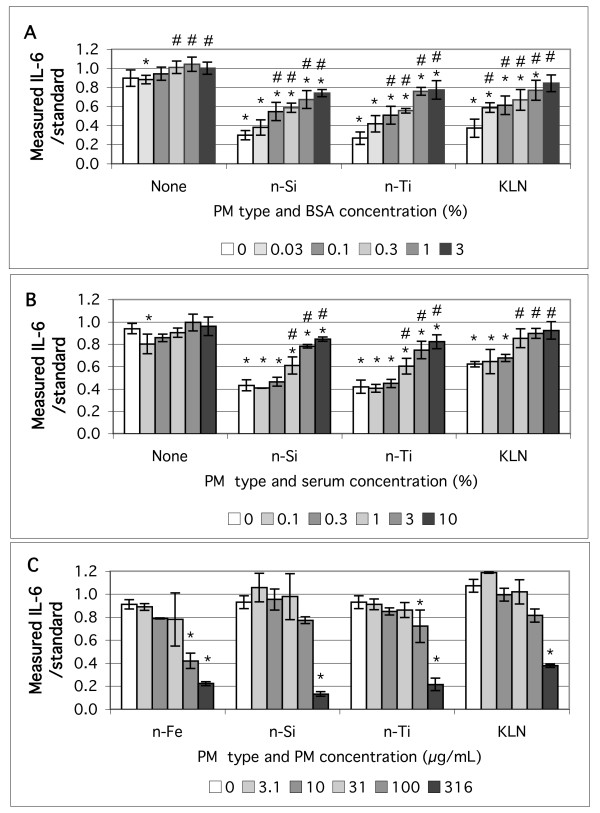
High surface area particles can interfere with measurement of cytokines in cell-free media containing a known aliquot of recombinant IL-6. A. Addition of 200 μg/mL of nano-SiO_2_, nano-TiO_2_, or kaolin to KGM media with the indicated concentration of BSA added. B. Addition of 200 μg/mL of nano-SiO_2_, nano-TiO_2_, or kaolin to KGM media with the indicated concentration of bovine serum added. C. The effect of adding increasing amounts of the indicated particles to KGM media without supplemental protein. Measured IL-6 divided by standard, * indicates significantly less than standard prepared in assay diluent, # indicates significantly greater than the IL-6 in the same particle suspension in media without exogenous protein addition, (two-tailed, p < 0.05). N = 3.

During initial experiments we observed apparent artifacts in the measured cytokine concentrations and hypothesized that surface adsorption on some particles reduced the ELISA measurements. Experiments were conducted in which a known amount of recombinant IL-6 was added to suspensions of particles in cell culture media and in media with supplemental protein. The data were consistent with non-specific binding of IL-6 to surfaces. Figure 6a and 6b show the effect of increasing exogenous protein supplementation using bovine serum albumin (BSA) or newborn calf serum on the measured IL-6 concentration in cell-free suspensions of kaolin, nano-sized SiO_2_, and TiO_2_. Without particles the measured IL-6 in cell culture media was close to the standard in serum-based assay diluent. The presence of 200 μg/mL of particles reduced the measured IL-6 concentration compared to the IL-6 standard in assay diluent, denoted by * in the graph. The measured IL-6 concentration increased with increasing amounts of supplemental protein. Addition of greater than 0.1% BSA and 1% FCS to the media resulted in a statistically significant increase in measured IL-6 compared to the same particle suspension without supplemental protein, denoted by # in the graph. Both the SiO_2 _and TiO_2_nanoparticles resulted in a statistically significant reduction in measured IL-6 compared to the standard for all mixtures including the highest tested protein supplementation: 3% BSA and 10% FCS. The highest level of protein supplementation eliminated the measurement artifact for 200 ug/mL of kaolin. The results presented were obtained with KGM media, but similar results were seen with LHC-9 media (data not shown).

Figure 6c shows the effect of adding logarithmically increasing concentrations of kaolin and nano-sized Fe_2_O_3_, SiO_2_, and TiO_2 _particles to cell culture media without supplemental protein. Particle concentrations of 3.1, 10, and 31 μg/mL did not cause a statistically significant decrease in measured IL-6. The p-values for 100 μg/mL of kaolin and nano-SiO_2 _were only slightly greater than 0.05, and the 316 μg/mL concentration of all particles resulted in a statistically significant decrease. The 100 μg/mL particle concentration corresponds to the 53 μg/cm^2 ^particle concentration which was used as the high treatment level in the cell response experiments (400 μL suspension in a 0.75 cm^2 ^well). Protein concentrations, measured by the Bradford assay and compared to a BSA standard, are 24 and 39 μg/mL for as-formulated KGM and LHC-9 media respectively. Media used for cell culture contains slightly higher protein due to cell secretions (2 – 4 μg/mL increase at 24 h). The use of 0.1% BSA in the cell culture media therefore corresponds to about a 50-fold increase in soluble protein available to block non-specific binding.

The wetted plastic area in the cell culture well is about 2.4 cm^2^. In contrast, at 100 μg/mL of particles and 400 μL of suspension per cell culture well the nitrogen adsorption surface area of the particles ranges from 19 cm^2 ^for kaolin to 210 cm^2 ^for nano-Al_2_O_3_, which is 1–2 orders of magnitude more than the wetted plastic surface area. Even if nitrogen adsorption area is a poor surrogate for the surface area available for aqueous protein adsorption, the difference in calculated area of the plastic and the particles supports the hypothesis that IL-6 adsorption on particle surfaces was the mechanism responsible for the measurement artifact.

**Figure 7:**
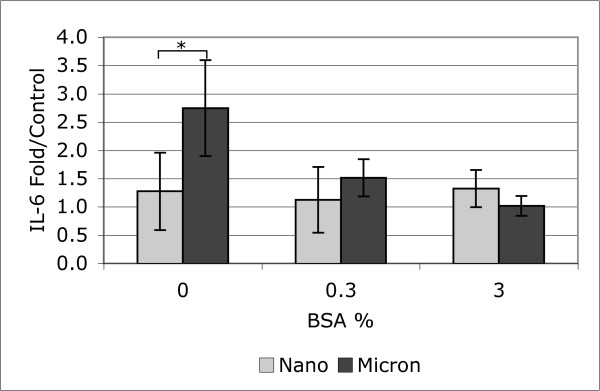
Response of BEAS-2B cells to SiO_2 _particles when treated in LHC-9 media with varying concentration of BSA. Data are merged from two independent experiments, mean ± s.d., N = 12, * indicates statistically significant difference between particle sizes.

Additional experiments were conducted with nano- and micron-sized SiO_2 _at higher concentration of BSA than used for the Figure 2 data. The data, Figure 7, show that the response of BEAS-2B cells to the SiO_2 _particles remains small, and this supports the conclusion that, with this *in vitro *model, pure oxide nanoparticles are not highly potent compared to either the soil-derived dusts or the positive controls. Figure 7 suggests that the measured rank order of response to the nano- and micron-sized particles may switch as the exogeneous protein is increased, but the difference between particle sizes did not reach statistical significance for the 3% BSA concentration. The control levels of IL-6 also showed a statistically significant increase with increased BSA: 3, 9, 15 pg/mL for zero, 0.3 and 3% BSA respectively. Also, 3% BSA appears to reduce the growth rate of the BEAS-2B cells relative to the as-formulated media.

### Endotoxin

**Figure 8:**
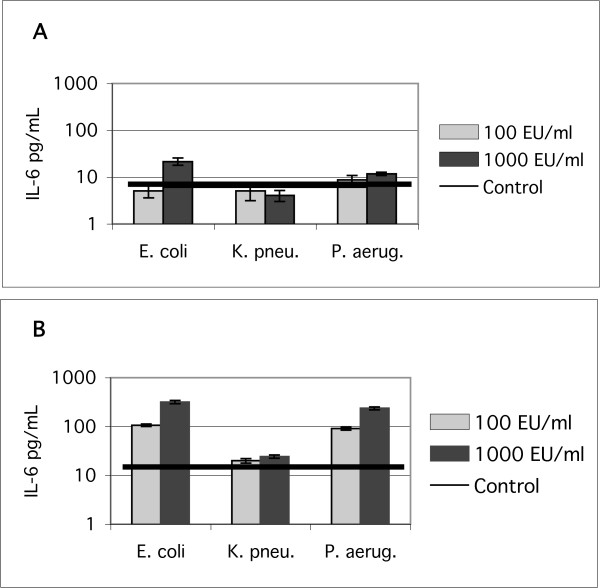
IL-6 response of BEAS-2B cells treated with the indicated concentrations of commercial lipopolysaccharide from three bacterial strains. A. in KGM media. B. in LHC-9 media. Mean ± standard error of the mean, N = 6–12 determinations.

We compared the relative potency of different commercial LPS preparations (Sigma) to verify that the low response to LPS was not due to the specific LPS type selected. Figure 8 shows the response of BEAS-2B cells in KGM (8a) and LHC-9 (8b) media to treatment with 100 and 1000 endotoxin units/mL (EU/mL) of commercial LPS from Escherichia coli 055:B5, Klebsiella pneumoniae, Pseudomonas aeruginosa serotype10. The E. coli and P. aeruginosa LPS had similar potency. The data also show that the response of BEAS-2B cells to all three tested types of LPS was higher when the cells were cultured in LHC-9 media compared to KGM.

### Cell-free ROS

**Figure 9:**
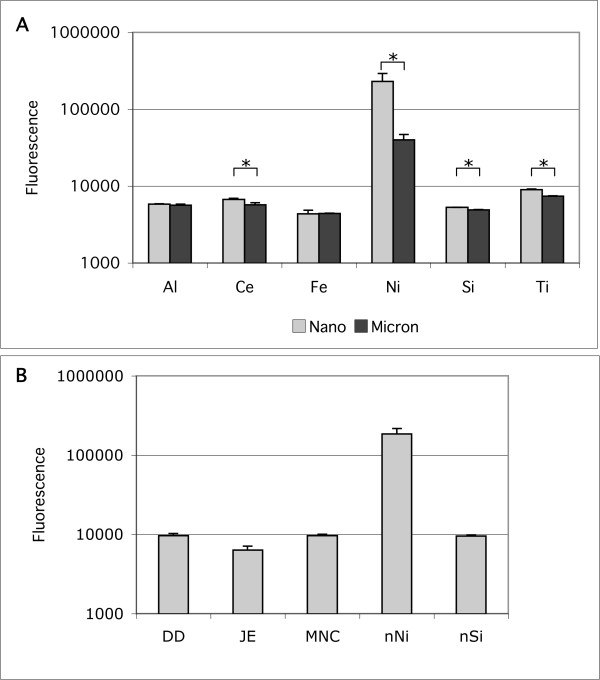
Reactive oxygen species produced by nano- and micron-sized metal oxide particles as measured by relative fluorescence in the cell-free DCFH assay. A. comparison of nano- and micron-sized particles of the indicated oxides. * indicates statistically significant difference between particle sizes for the pair. B. comparison of two nanoparticles to soil-derived dusts. Data are mean ± s.d., N = 3.

The potency to induce IL-6 in BEAS-2B cells does not correlate with the extracellular generation of reactive oxygen species (ROS). Typical data for the generation of ROS by particles in cell-free culture media are shown in Figure 9. These data are measurements of the relative fluorescence 5 minutes after adding 300 μg/mL of particles to KGM media, and are typical of readings taken from 1 to 10 minutes after adding the probe. The nano-sized NiO produced the highest level of fluorescence from DCFH reagent oxidation. For CeO, NiO, SiO_2 _and TiO_2 _the nanoparticles produced statistically higher fluorescence than the micron-sized particles. Figure 9b compares the nanoparticles of NiO and SiO_2 _to the three soil derived dusts.

## Discussion

We used the *in vitro *responses of cultured human lung epithelial cells as a model system to study the potential inhalation health effects of several types of commercially available manufactured nanoparticles of metal and ceramic oxides. Similar *in vitro *airway epithelial cell models have been used to study many other types of environmentally relevant PM. Examples of studies with BEAS-2B cells include ambient particulate matter,[35] residual oil fly ash,[31,33,36] wood smoke and Mt St. Helens ash, [37] and tire and pavement wear particles. [38] Examples of work with A549 cells includes TiO_2 _and Fe_2_O_3_,[10] urban PM, [39] road and quarry dust [6,40] and swine barn dust. [41]*In vitro *models are ideal for studying molecular mechanisms of toxicity because of the ease of manipulation and because the simplified biological system is not confounded by regulatory processes acting in the whole animal. Studies of this type are frequently designed to study specific toxicological mechanisms by inducing a strong response that can be ameliorated by cotreatment with antagonists.

The small difference between equal mass doses of nano- and micron-sized particles of the same nominal substance and the general low level of response to the oxide particles were contrary to the initial study hypothesis. Further investigation into molecular mechanisms of oxide nanoparticle interaction with airway cells will require an alternative *in vitro *model because the responses of BEAS-2B cells to nanoparticles were small. Our observation of small size-dependent differences may be due to either the low potency of the pure oxide particles or the characteristics of the BEAS-2B cells.

The pure metal oxides may not be highly potent for inducing proinflammatory cytokine signaling responses in lung epithelial cell lines. If true, this is good news for the manufacturers and users of oxide nanoparticles. Most *in vitro *reports of high potency for nanoparticles have involved either carbon-rich particles [42,43] or macrophage-like cell types such as RAW264.7, [44] J774A.1, [43] or THP-1 [45]. However, it is possible that the immortalized BEAS-2B epithelial cells do not respond to particles in the same way as tissues in a whole animal. The widely assumed hypothesis that nanoparticles are more potent is based largely on the seminal animal exposure studies with carbon black [46] and TiO_2_[47]. Caution is needed when comparing the rank order of potency of particles *in vitro *and *in vivo*, as was illustrated in a comparison of gasoline and diesel engine particles. [48]

We used crystalline silica (Min-U-Sil) as a prototype supermicron particle with known lung-damaging effects, but saw low cytotoxicity and IL-6 response. In seven experiments where control levels of IL-6 were well within the limit of detection the fold increase in response to a 50 μg/cm^2 ^treatment with Min-U-Sil ranged from 1.8- to 7.3-fold. The literature reports on the response of cultured cells to specific particle types, such as silica, shows complex and somewhat inconsistent patterns. Our observed response to Min-U-Sil and TiO_2 _can be compared to the results of Steerenberg et al., [49] who reported no increase over control for IL-6 in BEAS-2B cells treated with TiO_2_, comparable responses for diesel particles and α-quartz (17 ± 16 and 11 ± 15 fold), and higher response (59 ± 57 fold) response to fumed silica. Steerenberg's results suggest that different silica preparations have different potency, and that the IL-6 response of BEAS-2B cells is highly variable as indicated in Steerenberg's data where the standard deviation of IL-6 was comparable to the mean. A study by Xu et al. showed an inverted dose-response in A549 cells treated with Min-U-Sil with the 0.039 mg/mL treatment giving higher response than the 0.62 or 2.5 mg/mL treatments [50]. Xu also reported standard deviations approaching 50% of the mean for IL-6 response. In another study, DQ12 quartz was more cytotoxic than silicasol particles (60 and 100 nm) and neither quartz or silicasol nanoparticles induced IL-6 in A549 cells. [45] Micron-sized TiO_2 _is sometimes considered an inert particle and used as a negative control in particle studies, [30,49] but other studies have shown that nano-TiO_2 _can be proinflammatory *in vivo *[47,51] and can be taken up by nonphagocytic mechanisms by macrophages *in vitro *[52].

The comparisons of nano- and micron-sized oxide particles with environmental dusts provide an important context to interpret the cytokine results. Taken in isolation, our data show patterns, for example nano-sized silica inducing a statistically significant response compared to the micron-sized silica. However, as shown in Figures 2c and 2d and Figure 3 the IL-6 secretion of BEAS-2B cells treated with all the pure oxide particle types is low compared to the response to both the positive controls and some samples of soil-derived dust, a ubiquitous pollutant that is often considered benign. Further, the response of BEAS-2B cells to soil-derived dust is comparable to, or smaller than, the response of these cells to soluble metal salts such as synthetic residual oil fly ash (ROFA) used in our work [30] and in many studies by the US EPA [31-34,53,54] and others. The relative potency of the metal oxide, environmental, and soluble metal-rich materials suggests the possibility that most of the variation shown in Figures 2a and 2b consists of artifacts occuring near the control level of IL-6 and not robust oxide particle-induced signaling responses relevant to tissue inflammation.

The low potency of the metal oxides is consistent with our previous work showing that IL-6 secretion by BEAS-2B cells is correlated to the low volatility organic components [30] and that heating soil-derived dust to 300–550°C removes the potent components from soil-derived dusts. We have previously considered the possibility that the potent factor in the soil-derived dusts is endotoxin. Evidence includes the poor correlation between the potency for inducing IL-6 and the endotoxin content [30], and the observation that the response to the soil-derived particles is much greater than the response to commercial endotoxin applied at much higher concentration than the measured endotoxin in the dust sample. [29] The rank order of IL-6 secretion and endotoxin content for the DD, JE and MNC samples correlate, but the correlation of IL-6 with endotoxin was weaker in a larger group of 28 samples giving an R^2 ^= 0.43 [30]. As seen in Table 2, the potency rank order of the three soil-derived dusts correlates with the both the endotoxin content and the organic carbon content, supporting the hypothesis that other organic compounds besides endotoxin may be the potent factor(s).

The extracellular generation of ROS by surface-catalyzed reactions [55-58] has been proposed as a mechanism by which particles may damage biomolecules [20] or activate cell-surface receptor proteins. [59,60] In this study the potency for induction of IL-6 secretion did not correlate with extracellular ROS. The lack of correlation between ROS and cytokine response is similar to the results of Ovrevik et al. in a study of mineral particles and iron content. [61] A study with NHBE cells treated with coarse, fine, and ultrafine ambient particles found that the coarse PM was more potent for inducing cytokines but not intracellular ROS. [62]

Caution is needed in generalizing from these *in vitro *results to human exposures because of effects associated with the particular biological model used for the experiments. Because cells *in vitro *are isolated from the chemical and neural signaling that takes place in a whole organism there is no *a priori *reason to expect any one cell model to be the best surrogate for tissues *in vivo*. In a concurrent project we have found that different combinations of cell line, culture media, and passaging protocol details can change the cytokine secretion response to a given particle treatment [Manuscript in preparation]. We therefore reported the results of the same particle treatments used in several different cell models, and further compared the manufactured oxide particles to other particles previously tested using the same cell line and protocol.

High surface area materials can adsorb chemicals from cell culture media and therefore confound data from in vitro toxicology experiments. Adsorbtion of cytokines has been shown by both the work of Seagrave et al. [63] with IL-8 in the presence of carbon black, and our experiments with IL-6 in the presence of oxide particles. Carbon particles have also been reported to adsorb indicators, such as neutral red and MTT, from the media introducing artifacts into viability assays. [64] Particle surfaces can have indirect effects on cells *in vitro *by adsorbing trace nutrients or growth factors from the finite volume of culture media and making these vital factors less available to the cells. [64]

A key confounding issue is the extent that the cytokine adsorbtion artifact presented in Figures 6 and 7 is responsible for the low potency of the oxide particles when compared to soils and the small differences between the nano-sized and micron-sized particles. Figure 7 suggests that the rank order may be influenced by the adsorbtion artifact, but this is a qualitative trend that did not reach statistical significance. The experiments quantifying the effect of supplemental protein on cell-free measurements of IL-6 indicated that the particle concentration used for the cell treatments was borderline for causing a statistically significant reduction in measured IL-6. Combined, this evidence suggests that the low responses to metal oxide particle treatments reported in this study are a real lack of cell activation and not the result of a false negative artifact. Given the general low level of *in vitro *BEAS-2B cell response to the oxide particles compared to the environmental particles, it may be more productive to do further study of particle size differences in another cell model and measuring other endpoints. However, the possibility that nanoparticles, and other high-surface-area particles can cause non-biological artifacts should be considered in the design of future experiments.

## Conclusion

This study, using lung epithelial cells *in vitro*, indicates that manufactured particles of Al, Ce, Fe, Ni, Si, and Ti oxides occasionally induced statistically significant increases in the secretion of the proinflammatory cytokines IL-6 and IL-8, but these responses were not robust and were small compared to certain soil-derived environmental dusts and positive controls. We tested the hypothesis that nano-sized metal oxide particles are more potent than micron-sized particles of the same nominal substance, but results were inconclusive due to the low responses. The changes in cytokine secretion by the oxide particle-treated cells are detectable, but may not be biologically important because the particle-induced responses are comparable to passage-to-passage variation in control levels and are small compared to the effects of other potential agonists such as TNF-α, a cytokine produced by macrophages in response to particles *in vivo*. Animal studies with carbon particles continue to report inflammatory responses that correlate with surface area [65], so the lack of a consistent size-dependent response in this study may reflect either a difference between inorganic oxide versus carbon/organic particles or a difference between cell culture and whole animal responses. High concentrations of suspended particles, especially nano-sized particles, can interfere with ELISA measurements of cytokine secretion, and this should be considered in both interpretation of the published literature and in the design of future experimentals.

This study adds to the growing literature on the biological effects of nanomaterials. While inhalation exposure to any mineral dust should be minimized, this *in vitro *study suggests that manufactured metal oxide nanoparticles are not exceptionally potent for causing proinflammatory signaling in airway epithelial cells when compared to more conventional ambient and occupational dusts.

## Methods

### Materials

The nano-sized and micron-sized particles and the positive controls were purchased from commercial suppliers indicated in Table 1. The three soil-derived dusts DD, JE, and MNC were derived from field samples collected on an unpaved desert road in Utah, on an urban street in Ciadud Juàrez, Mexico, and from native soil at a remote desert site in Utah respectively. The DD, JE, and MNC particles are identical to samples 18, 16, 28 respectively in [30] which deals with the correlation between chemical composition of soil-derived dusts and IL-6 induction in BEAS-2B cells. The samples DD and MNC also correspond to UTDG and UTMC in [66] which contains detailed composition data and describes the procedures used for chemical analysis. The field samples were resuspended in the laboratory, and the particles were aerodynamically separated to provide a PM_2.5_-enriched material for the cell treatments as previously described [30,67].

### Surface area

Particle surface area per mass was measured by nitrogen adsorption (BET single point method) using a Quantachrome Monosorb analyzer. Surface determinations were run in duplicate for both the adsorption and desorption periods. Surface mean diameter was calculated assuming spherical particles and the mineral density from handbooks.

### Cell culture

Four different cell culture models were used: the immortalized cell line BEAS-2B in LHC-9 media, BEAS-2B cells in KGM media, A549 cells in DMEM/F12, and normal human bronchial epithelial cells (NHBE) in BEGM media. Table 3 summarizes the details of the protocols used for the different cell culture models. The generic cell culture protocol consisted of growing the cells in an incubator at 37°C/6% CO_2 _in 75 or 150 cm^2 ^flasks, replacing media every 2–3 days, and passaging before confluence by dislodging with trypsin, washing, and seeding new flasks or treatment wells. The use of LHC-9 media for BEAS-2B cells was based on the original work of Lechner et al. [68], particle studies by other laboratories, for example [69], and our prior studies. [29,30] The protocol for BEAS-2B cells in KGM was based on the methods originally developed by the US EPA and since used in multiple studies [31,36,70,71]. The protocol for the NHBE cells was based on the supplier's recommendation. Particle experiments consisted of seeding 48-well plates (Costar, Fisher Scientific) with cells, allowing a recovery period for the cells to attach and proliferate, replacing the culture media with fresh media containing the treatments, and harvesting the media for cytokine analysis 24 hr after treatment. Culture media were used both as formulated and with 0.1% added bovine serum albumin.

**Table 3:** Cell culture methods.

Cells	BEAS-2B	BEAS-2B	NHBE	A549
Supplier	US EPA	US EPA	Cambrex	ATCC
Culture Media	KGM	LHC-9	BEGM	DMEM/F12 10% FBS
Supplier	Clonetics CC-3001 with CC-3111 kit	Biosource	Clonetics	ATCC
Precoat on culture surface	No	No	Collagen/fibronectin	No
Seeding in treatment wells	35,000/cm^2^	35,000/cm^2^	2500/cm^2^	20,000/cm^2^
Seeding to PM treatment	3 days	3 days	7 days	3 days
Passage Number	75–85	75–85	5–7	80–82

### Particle treatment

Particles were weighed, mixed with cell culture media, and resuspended by sonication and vortexing immediately before adding to the cell wells. The treatment experimental design consisted of multiple treatments applied to cells from a single passage to minimize confounding of comparisons by passage-to-passage variation of the cultured cells. Each multiwell cell culture plate included positive and negative controls. Results were replicated using additional independent passages of cells. Particle treatment concentrations were 0.53, 5.3 and 53 μg/cm^2 ^which correspond to 1, 10 and 100 μg/mL respectively for the cell culture plates and media volumes used.

### Cell viability

Viable cell count was measured using the Cell Counting Kit -8 assay from Dojindo Laboratories. This assay assumes that the relative number of cells is linear with the metabolic activity indicated by mitocondrial reduction of WST-8 (2-(2-methoxy-4-nitrophenyl)-3-(4-nitrophenyl)-5-(2,4-disulfophenyl)-2H-tetrazolium, monosodium salt) to produce a water-soluble product. Data are quantified by absorption at 450 nm, corrected by absorption at 650 nm, using a Molecular Devices plate reader.

### Cytokine protein secretion

The cytokines IL-6 and IL-8 were measured using an enzyme-linked immunosorbent assay (ELISA). Antibodies, cytokine standard, and avidin horseradish peroxidase (AVHRP) for IL-6 were obtained from eBioscience; the IL-8 assay used a Human CXCL8/IL-8 DuoSet kit (DY208) from R&D Systems. Nunc MaxiSorp immuno plates (Fisher Scientific) were coated overnight with antibody; room-temperature incubations on a plate rocker were carried out with sample and standards, biotin-conjugated antibody, and AVHRP with thorough washing with 0.05% Tween-20 in PBS pH 7.0 between steps. After a 1-hour incubation with ABTS substrate, the plates were read at 405 nm using a Molecular Devices plate reader with SoftMax Pro software, and cytokine concentrations were calculated against a standard curve prepared in duplicate.

### Cell-free artifacts of high-surface PM

The experiments to quantify particle artifacts in the cytokine analysis used the addition of known amounts of recombinant human IL-6 from R&D Systems to various aqueous phases including water, phosphate buffered saline, LHC-9 or KGM cell culture media, cell culture media supplemented with serum or BSA, and media conditioned by growing cells for 24-h. Mixtures of IL-6 in the aqueous phase were incubated alone or with particles, centrifuged to separate the particles, decanted, and stored frozen until analyzed by ELISA. Cytokine quantification was by both direct comparison of measured concentrations in particle-free versus particle-treated samples and by a standard curve using recombinant IL-6 in serum-based assay diluent and prepared in duplicate at the time of analysis. Most cell-free experiments used concentrations similar to typical cell culture experiments, for example, 100 μg/mL particles, 200 pg/mL IL-6. However, the hypothesis-testing experimental conditions were varied over a wide range using logarithmically spaced nominal values: particles from zero to 400 μg/mL, IL-6 from zero to 10,000 pg/mL, incubation time from 5 minutes to overnight.

### Endotoxin

Endotoxin was measured using the chromogenic Limulus Amebocyte Lysate (LAL) assay kit QCL-1000 (Cambrex Biosciences).

### Cell-free ROS

The generation of reactive oxygen species in cell-free media was determined using the dichlorodihydrofluorescein diacetate (DCFH-DA) reagent (Molecular Probes #D-399). The method was based on studies of extracellular ROS generation by silica [57]. For cell-free experiments the acetate was cleaved to form the non-fluorescent precursor by treatment with 0.1 M NaOH followed by neutralization and dilution to working concentration. Dichlorodihydrofluorescein (DCFH) is converted to the fluorescent product dichlorofluorescein by oxidation. Particles were added to 10 μM DCFH at 10–1000 μg/mL final concentration, incubated, and read at fixed intervals starting immediately after DCFH addition. Data in Figure 9 were from the third reading, nominally starting at 5 minutes. Fluorescence was read on a Perkin Elmer Victor 3 V Multilabel counter using 485 nm excitation and 535 nm emission. Freshly diluted hydrogen peroxide was used as a positive control.

### Statistics

Data were analyzed with JMP software (SAS Institute). Paired comparisons were made using Student's t-test, comparison of multiple treatments to a common control used one-way ANOVA with Dunnett's test, and p < 0.05 was considered significant.

## Competing interests

The author(s) declare that they have no competing interests.

## Authors' contributions

The study design, statistical analysis, and preparation of the manuscript were by JMV, cell culture experiments and ELISA assays were conducted by MK, EGK, and MMV, and data base assembly was by EGK. The diagnosis of the particle artifacts was by MMV. GSY provided advice, infrastructure, and assistance in preparation of the manuscript.
